# Comparative Advantages of Ether and Chloroform

**Published:** 1859-12

**Authors:** 


### Comparative Advantages of Ether and Chloroform.

In a discussion which lately took place in the New York Academy of Medicine, on the comparative merits of ether and chloroform, as Anæsthetic agents, which we find detailed in the Philadelphia *Medical and Surgical Reporter*, some interesting remarks were made by Prof. Dalton. Coming from so eminent a physiologist, they are, we conceive, entitled to much authority. The opinion of Dr. Dalton, as to the cause of death, in fatal cases of inhalation of chloroform, is that this agent produces paralysis of the heart. To this conclusion he is led by observing the effects of ether and chloroform on animals who are caused to inhale them. If the process is carried to a moderate extent, and the chest of the animal be then opened as quickly as possible, the heart will continue to beat for a considerable length of time. If, however, the inhalation be pushed until respiration is stopped, it will be found, on opening the chest, that though the heart be still beating, its movements are very feeble. If anæsthesia be carried only to the stoppage of respiration, the animal usually recovers; but if when the respiration ceases, the heart also is still, the animal never recovers.

Now, although fatal results have occurred in Dr. Dalton's hands, in experimenting on animals, with both ether and chloroform, when inhalation is pushed to the extreme, he has been obliged to take a great deal of pains to produce this effect with ether, whereas death often follows the use of chloroform notwithstanding the best precautions. In fact, he has for this reason altogether abandoned the use of chloroform, and instead of it employs sulphuric ether. "I think I may say," he remarks, "without exaggeration, that I am thoroughly convinced that there is a radical difference in the danger following the administration of these two substances. I am sure that chloroform is more dangerous to animals, at least." Dr. Dalton also stated, in reply to the question from the president, Dr. Watson, whether he had found ether fail to produce all the effects that can be wished for from chloroform, that it had never failed his hands, and his experience would lead him to believe that it is capable of producing all the effects of chloroform.

We confidently expect that a reaction will soon take place in the mind of the medical public on this important subject. The fact that no well-authenticated case exists of death which can be attributed to the inhalation of ether, and that this agent is capable of producing all the effects, as an anæsthetic, which chloroform can, must ere long cause its universal substitution for the latter dangerous agent.—*Boston Medical & Surgical Journal.*

